# A new class of IMP dehydrogenase with a role in self-resistance of mycophenolic acid producing fungi

**DOI:** 10.1186/1471-2180-11-202

**Published:** 2011-09-16

**Authors:** Bjarne G Hansen, Hans J Genee, Christian S Kaas, Jakob B Nielsen, Torsten B Regueira, Uffe H Mortensen, Jens C Frisvad, Kiran R Patil

**Affiliations:** 1 Center for Microbial Biotechnology, Department of Systems Biology, Technical University of Denmark, 2800 Kgs Lyngby, Denmark; 2 Novozymes A/S, Krogshoejvej 36, 2880 Bagsvaerd, Denmark; 3 Structural and Computational Biology Unit, EMBL-Heidelberg, Meyerhofstrasse 1, 69117 Heidelberg, Germany

## Abstract

**Background:**

Many secondary metabolites produced by filamentous fungi have potent biological activities, to which the producer organism must be resistant. An example of pharmaceutical interest is mycophenolic acid (MPA), an immunosuppressant molecule produced by several *Penicillium *species. The target of MPA is inosine-5'-monophosphate dehydrogenase (IMPDH), which catalyses the rate limiting step in the synthesis of guanine nucleotides. The recent discovery of the MPA biosynthetic gene cluster from *Penicillium brevicompactum *revealed an extra copy of the IMPDH-encoding gene (*mpaF*) embedded within the cluster. This finding suggests that the key component of MPA self resistance is likely based on the IMPDH encoded by *mpaF*.

**Results:**

In accordance with our hypothesis, heterologous expression of *mpaF *dramatically increased MPA resistance in a model fungus, *Aspergillus nidulans*, which does not produce MPA. The growth of an *A. nidulans *strain expressing *mpaF *was only marginally affected by MPA at concentrations as high as 200 μg/ml. To further substantiate the role of *mpaF *in MPA resistance, we searched for *mpaF *orthologs in six MPA producer/non-producer strains from *Penicillium *subgenus *Penicillium*. All six strains were found to hold two copies of IMPDH. A cladistic analysis based on the corresponding cDNA sequences revealed a novel group constituting *mpaF *homologs. Interestingly, a conserved tyrosine residue in the original class of IMPDHs is replaced by a phenylalanine residue in the new IMPDH class.

**Conclusions:**

We identified a novel variant of the IMPDH-encoding gene in six different strains from *Penicillium *subgenus *Penicillium*. The novel IMPDH variant from MPA producer *P. brevicompactum *was shown to confer a high degree of MPA resistance when expressed in a non-producer fungus. Our study provides a basis for understanding the molecular mechanism of MPA resistance and has relevance for biotechnological and pharmaceutical applications.

## Background

**Figure 1:**
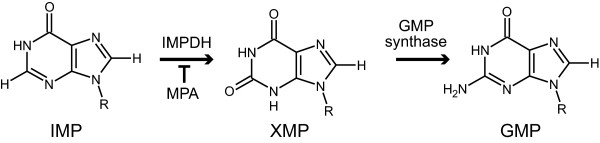
**Role of IMPDH and MPA in GMP biosynthesis**. MPA inhibits IMPDH. MPA: Mycophenolic acid. R: ribose 5'-monophosphate. IMP: inosine-5'-monophosphate, XMP: xanthosine-5'-monophosphate, guanosine-5'-monophosphate. GMP: Guanosine monophosphate. IMPDH: IMP dehydrogenase.

Mycophenolic acid (MPA) is the active ingredient in important immunosuppressive pharmaceuticals such as CellCept^® ^(Roche) and Myfortic^® ^(Novartis). The target of MPA is inosine-5'-monophosphate dehydrogenase (IMPDH) [1], which catalyses the conversion of IMP to xanthosine-5'-monophosphate (XMP). This reaction is the first committed and the rate-limiting step in guanine nucleotide biosynthesis [2] (Figure 1). The ability to produce MPA is almost exclusively found in species from the *Penicillium *subgenus *Penicillium*, where several species have been reported to produce MPA [3]. The fact that producer fungi are resistant towards their own toxic metabolite (in this case MPA) suggests the presence of metabolite-specific resistance mechanisms [4,5]. Several fungal secondary metabolites have medical applications - ranging from antibiotics to immunosuppressants. Thus, elucidation of the underlying molecular mechanisms of self-resistance in producer fungi is of great interest for biotechnological as well as health applications. For example, efficient production of drugs in a microbial cell factory may greatly depend on increasing the tolerance of the host organism to the drug. In the few published examples in eukaryotes, self-resistance to bioactive secondary metabolites has been attributed to either presence of an enzyme modifying the compound [6,7], export mechanisms [8,9], compartmentalization [10], or a few specific mutations in the target enzyme [11]. In the case of MPA, the self-resistance mechanism has not been elucidated.

The MPA biosynthetic gene cluster from *Penicillium brevicompactum *was identified only recently [12]. Interestingly, it turned out that the MPA gene cluster, in addition to the MPA biosynthetic genes, contains a putative IMPDH-encoding gene (*mpaF*). The study also revealed an additional putative IMPDH-encoding gene by probing the *P. brevicompactum *genomic DNA [12]. A BLAST search using *mpaF *as query resulted in only a single IMPDH encoding gene per organism for all fully sequenced non-*Penicillium *filamentous fungi (see the Results and Discussion section for details). Thus, the discovery of *mpaF *identifies *P. brevicompactum *as the first filamentous fungus known to feature two IMPDH encoding genes. In this study, we have identified additional species from the *Penicillium *subgenus *Penicillium *that contain two putative IMPDH encoding genes. Furthermore, we show that the two copies that are present in each fungus are dissimilar, and that one of them forms a new distinct group in a cladistic analysis. The IMPDH from the MPA cluster, *mpaF*, is the founding member of this novel group. The presence of *mpaF *within the biosynthesis cluster in *P. brevicompactum *hints at a role in MPA self-resistance. In this study, we examine this hypothesis and show that *mpaF *confers resistance to MPA when expressed in an otherwise highly sensitive non-producer fungus *Aspergillus nidulans*.

## Results and discussion

### Expression of *mpaF *in *A. nidulans *confers resistance to MPA

**Figure 2:**
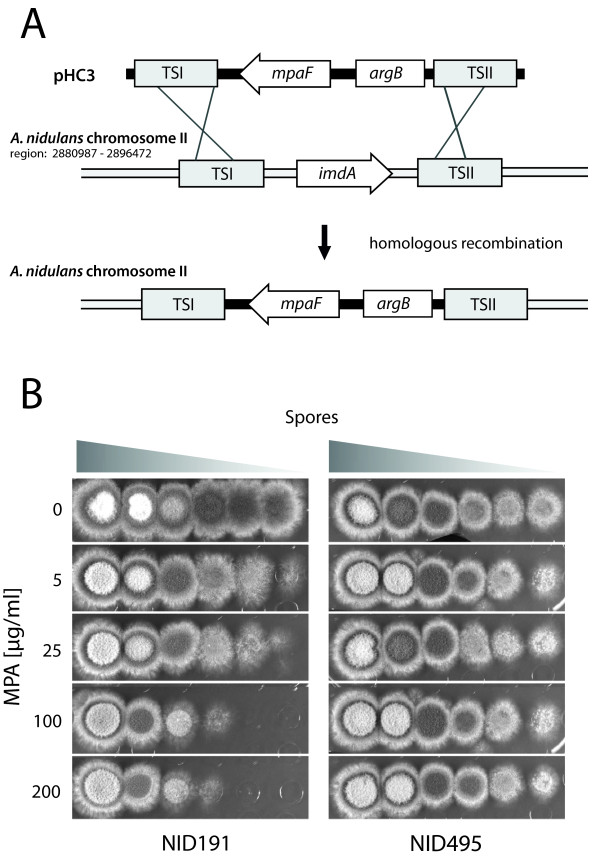
**MpaFp confers resistance towards MPA**. A) Replacing native IMPDH-A coding gene (AN10476, *A. nidulans imdA*) with *mpaF *by homologous recombination. The gene targeting substrate contains four parts: *mpaF *(IMPDH from MPA gene cluster), *argB *(selection marker) and finally TSI and TSII (targeting sequence I, 2197 bp; and II, 2244 bp flanking AN10476 (*A. nidulans *IMPDH)). B) Spot assay to determine sensitivity towards MPA. Ten-fold serial dilutions of spores from the two strains NID191 (reference strain with native *A. nidulans imdA*) and NID495 (*A. nidulans imdA *replaced with *mpaF*) were spotted on minimal medium plates with 0, 5, 25, 100 and 200 μg MPA/ml. Each row is composed of spots containing plated spores ranging from ~10^6 ^(to the left) to ~10 (to the right) as indicated in the figure.

In order to investigate whether MpaFp from *P. brevicompactum *is resistant to MPA we transferred *mpaF *to a fungus, *A. nidulans*, which does not produce MPA. Specifically, we constructed a strain where the *A. nidulans *IMPDH structural gene (*imdA*) was replaced by the coding region of *mpaF*, see Figure 2A. The sensitivity of this strain towards MPA was then compared to a reference *A. nidulans *strain. As expected, the spot assays shown in Figure 2 demonstrate that the germination of WT spores is reduced due to MPA. This effect is most significant at media containing 100 and 200 μg/ml MPA where the viability is reduced by approximately two orders of magnitude as compared to the plate containing no MPA. The level of sensitivity of *A. nidulans *towards MPA is consistent with the toxic levels observed for other eukaryotic organisms [13,14]. In contrast, MPA had little or no effect on spore viability of the strain NID495 where the gene encoding *A. nidulans *IMPDH (*imdA*) has been replaced by *mpaF*. Accordingly, we conclude that *mpaF *encodes an IMPDH, which is functional and less sensitive to MPA as compared to the IMPDH encoded by *imdA*. Notably, this degree of resistance has previously been observed only for IMPDH proteins of prokaryotic origin [1].

### A new class of IMPDHs found in the *Penicillium *subgenus *Penicillium*

The data above strongly suggest that *mpaF *encodes an IMPDH, which is resistant to MPA, hence strengthening the hypothesis that the IMPDH-encoding gene residing within the MPA gene cluster plays a distinctive role in MPA self-resistance. The results also lead to the next question - whether only MPA producers have two copies of IMPDH-encoding genes. We first performed a BLASTx search (default settings, August 2010, see Methods) by using the cDNA sequence of *mpaF *as a query. Two IMPDH-encoding genes from *Penicillium chrysogenum*, the only *Penicillium *species with a publicly available sequenced genome, produced the most significant hits (data not shown). As *P. chrysogenum *is not able to produce MPA, the presence of two IMPDH-encoding genes in this fungus is intriguing. Interestingly, the BLASTx search only revealed one IMPDH in the other filamentous fungi that have their genome sequence available in the public domain. *Penicillium marneffei*, another *Penicillium *species included in the search, was found to contain only one IMPDH-encoding gene in its genome. However, even though *P. marneffei *is named a *Penicillium*, it is only distantly related to *Penicillium sensu stricto *[15]. Thus, the only two fungi known to have two IMPDH copies so far are the *Penicillium *species, *P. brevicompactum *and *P. chrysogenum*. An initial cladistic analysis showed that the *P. brevicompactum *IMPDH protein encoded by *mpaF *and one of the two IMPDHs from *P. chrysogenum *are phylogenetically highly distinct from the other IMPDHs from filamentous fungi. Furthermore, the IMPDH-encoding gene from *P. brevicompactum *that was not located within the MPA gene cluster and one of the two IMPDH-encoding genes from *P. chrysogenum *clustered together with the IMPDH-encoding genes from *Aspergillus *species (data not shown). Notably, this group was distinct from the group containing *mpaF*. Accordingly, we decided to name the original and *mpaF *types of IMPDH as IMPDH-A and IMPDH-B, respectively. The classification of IMPDHs was further substantiated with IMPDH sequences obtained from more *Penicillium *species as described in the following.

**Figure 3:**
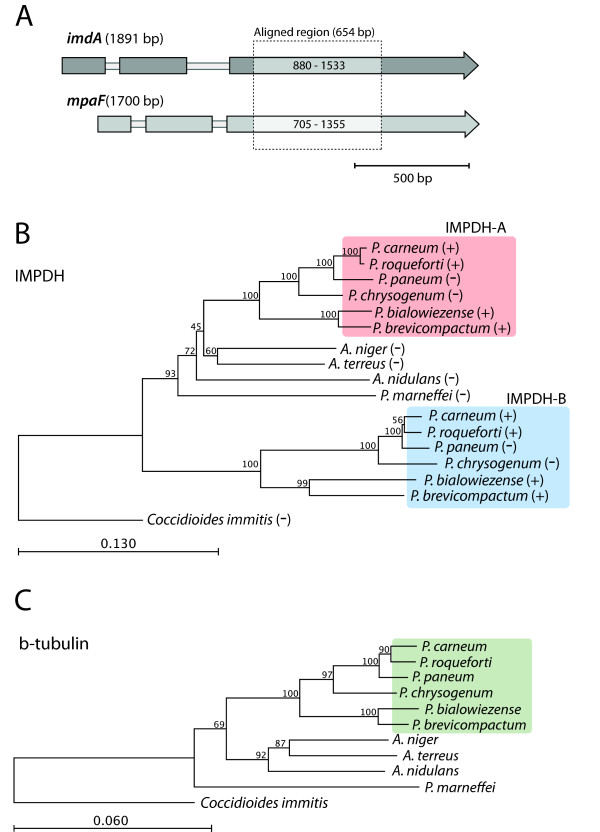
**Identification and cladistic analysis of IMPDH-A and IMPDH-B coding genes from different fungi**. A) Gene organization of *imdA *from *A. nidulans *and *mpaF *(coding for IMPDH-B in *P. brevicompactum*). The sequence region used for creating the cladograms in B is marked by a square. Introns are marked by a thin open line. B) and C): Rooted cladograms based on, B) IMPDH cDNA sequences (651-654 bp); and C) *β-tubulin *cDNA sequences (981 bp) from species from *Penicillium *subgenus *Penicillium *and from five fungi with sequenced genomes including the outgroup. *P*.: *Penicillium *and *A*.: *Aspergillus*. Bootstrap values (expressed as percentage of 1000 replications) are shown at the branch points. MPA production is indicated by "+" or "-". The clades with *Penicillium *subgenus *Penicillium *genes are boxed; red, IMPDH-A; blue, IMPDH-B; green, *β-tubulin*. *Coccidioides immitis *has been used as outgroup in both analyses B and C. Scale bars correspond to 0.130 and 0.060 nucleotide changes per site in cladograms B) and C) respectively.

*P. brevicompactum *and *P. chrysogenum *belong to *Penicillium *subgenus *Penicillium *and are closely related [16]. To investigate if the presence of two IMPDHs is a general phenomenon in *Penicillium *subgenus *Penicillium*, we created degenerate primers designed to amplify the genes coding for the two types of IMPDHs, IMPDH-A and IMPDH-B. These primers were used to amplify IMPDH-encoding genes by using gDNA from four additional *Penicillium *strains as PCR templates (Table 1). Interestingly, despite the fact that strains tested included both MPA producers and non-producers, we found two IMPDH copies in all four strains (Table 1). We then performed a cladistic analysis including these new genes, which showed that *mpaF *and its orthologs clearly form a separate group (Figure 3).

**Table 1:** Strains and sequences

Taxon name	IBT number	Other collection numbers	MPA prod.*	Sequences (Accession #)		
				
				*IMPDH-A*	*IMPDH-B*	*β-tubulin*
*P. bialowiezense*	21578	CBS 112477	++	JF302658	JF302662	JF302653
*P. brevicompactum*	23078	-	++	JF302657	HQ731031^+^	JF302652
*P. carneum*	3472	CBS 466.95	++	JF302656	JF302660	JF302650
*P. chrysogenum*	5857	NRRL 1951	-	XM_002562313	XM_002559146	XM_002559715
*P. paneum*	21729	CBS 112296	-	JF302654	JF302661	JF302651
*P. roqueforti*	16406	NRRL 849	+	JF302655	JF302659	JF302649

*) MPA production on CYA media. - means no production, + medium and ++ high production (Frisvad and Samson, 2004)).

^+^) The MPA gene cluster sequence from *P. brevicompactum *which contains the gene encoding MpaFp.

### Differences between IMPDH-A and IMPDH-B with a potential role in MPA resistance

**Figure 4:**
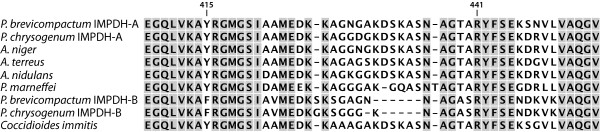
**Multiple sequence alignment of selected fungal IMPDHs**. The region including the amino acid residue at position 415 and part of the flap-region (flap-region being spanned by residues 412 - 467) is presented in the figure. The position 415 is tyrosine in all IMPDHs identified prior to this work [1]. Note that the flap region is very variable, with only residue 415Y and key catalytic residues 441R and 442Y completely conserved in all IMPDHs identified prior to this work [1]. Residues conserved among all nine sequences are highlighted in grey. *P. brevicompactum *IMPDH-B (encoded by *mpaF*) is used as a reference while referring to position numbers. *P, Penicillium; A, Aspergillus*.

We created a full-length alignment of IMPDH protein sequences from both MPA producers and non-producers to identify substitutions that might account for the extraordinary MPA resistance observed for heterologously expressed *P. brevicompactum mpaF *(type IMPDH-B). There are 30 residues known to be important for catalytic function and these are completely conserved in all IMPDHs identified at present [1]. All of the 30 residues, except for the one corresponding to position 415 (numbering follows MpaFp), were also conserved in IMPDH-B from both *P. chrysogenum *and *P. brevicompactum*. The residue at position 415 is part of the active site and was found to be phenylalanine in both IMPDH-B sequences (Figure 4); whereas this position is featured by tyrosine in all IMPDH-A type proteins. In addition, when comparing IMPDH-A and IMPDH-B sequences, the so-called IMPDH "flap-region" [1] is variable including a five-residue-long gap in the two IMPDH-Bs (Figure 4). Although these sequence differences may seem significant, they are not obvious candidates for conferring MPA resistance. The substitution at position 415 is not in close proximity to the MPA binding site and the sequence of the "flap-region" is known to be highly variable and has so far not been linked to MPA sensitivity [16]. Furthermore, *P. chrysogenum *is not a MPA producer and it is therefore not self-evident that the IMPDH-B from this fungus is resistant. Additional IMPDH sequences from MPA producers and non-producers will be useful in the search for the functionally critical residues. Moreover, comparative biochemical characterization of IMPDH-A and IMPDH-B, as well as of mutant derivatives, will be necessary to quantify the degree of resistance, and to pinpoint the residues important for MPA resistance. Such biochemical characterization, together with the measurement of expression levels of IMPDH-A and IMPDH-B in MPA producers, will help in dissecting the relative contribution of each type to MPA self-resistance.

### IMPDH-B has possibly emerged through gene duplication

IMPDHs are highly conserved enzymes, which points to their important role in fitness. A high level of conservation was also observed for the sequences obtained from the six *Penicillium *strains investigated in our study. In a cladogram containing both fungal and prokaryotic IMPDHs, the fungal genes of type IMPDH-B clearly cluster with the fungal IMPDH-A type and not the prokaryotic IMPDH-encoding genes (data not shown). The identity of the IMPDH-B to prokaryotic IMPDHs is ~30-35% on amino acid level. This relatively low degree of conservancy, combined with the fact that IMPDH-A and IMPDH-B are ~80% identical in filamentous fungi, suggests that the genes coding for IMPDH-B arose by gene duplication in a filamentous fungus, rather than via a horizontal gene transfer from a prokaryotic organism. As both IMPDH-A and IMPDH-B classes are present in all *Penicillium *subgenus *Penicillium *strains tested, a gene duplication event in *Penicillium *is a plausible hypothesis. To gather further support for this hypothesis, β-tubulin sequences were obtained for all of the fungi tested in this study and a cladogram based on β-tubulin sequences was calculated (Figure 3). As β-tubulin is a highly conserved protein with a critical functional role in eukaryotes, it is often used to create an organismal cladogram [16,17]. The cladogram based on IMPDH sequences is to a high degree in agreement with the β-tubulin cladogram, and both are in agreement with previously published β-tubulin cladograms [16]. Based on the observations from the cladograms and the high level of identity (~80%) between IMPDH-B type and IMPDH-A type sequences, we postulate that the IMPDH-encoding gene duplication took place during the divergence of *Penicillium *subgenus *Penicillium*, or earlier. The large genome sequencing effort that is being carried out at the moment may shed further light on the evolutionary origin of IMPDH-B.

## Conclusions

This is the first report elucidating the presence of a new class of IMP dehydrogenase, IMPDH-B, with a role in MPA resistance in filamentous fungi. The high level of resistance observed for IMPDH-B encoded by *mpaF *from *P. brevicompactum *is intriguing and stands as the strongest MPA tolerance reported for a eukaryotic IMPDH. Our study also provides insight into the possible molecular basis responsible for the high MPA resistance of IMPDH-B. In particular, we identified one amino acid residue, which is completely conserved in all previously identified IMPDHs, but which is different in the members of the group belonging to the type IMPDH-B. On the applied front, the identified genetic basis for self-resistance may help in efficient production of MPA in heterologous hosts and in understanding the MPA-related chemical ecology in filamentous fungi.

## Methods

### Strains and media

*A.nidulans *NID191 (*argB2, pyrG89, veA1, nkuA-trS::*AF*pyrG*, IS1::PgpdA-TtrpC::*argB) *[18] and NID495 (*argB2, pyrG89, veA1, nkuA-trS::*AF*pyrG*, Δ*imdA::argB::mpaF*) were grown on Minimal Medium (MM) containing 1% glucose, 10 mM NaNO_3_, 1 × salt solution [19], and 2% agar for solid media. MM was supplemented with 10 mM uridine, 10 mM uracil, and 4 mM L-arginine when necessary. *P. brevicompactum *IBT 23078 was grown on Czapek yeast autolysate (CYA) agar at 25°C. CYA: 5.0 g/l Yeast extract (Difco); 15 g/l agar; 35 g/l Czapek Dox broth (Difco); 10 mg/l ZnSO_4_·7H_2_O; 5 mg/l CuSO_4_·5H_2_O. The pH of the medium was adjusted to 6.5 by using NaOH/HCl.

### Generating expression construct

Amplification of DNA by PCR was performed using proof-reading PfuTurbo^® ^Cx Hotstart polymerase (Stratagene) in 50 μl according to the manufacturer's instructions. The reaction mixtures were heated to 95°C for 2 min followed by 30 cycles at 95°C for 30 s, 58°C for 30 s, and 72°C for 3 min. A fragment containing the fungal selection marker *argB *was amplified from the expression vector pU1111 [18] with primers BGHA71 and BGHA72 and cloned into MfeI/SbfI digested expression vector pU0002 [18] resulting in construct pHC1. A 2689 bp fragment containing *mpaF *including *mpaF *promoter and terminator was amplified using primers BGHA125 and BGHA132 from *P. brevicompactum *IBT 23078 gDNA and cloned into the KpnI/AsiSI site of pHC1 resulting in pHC2. The flanking regions of *imdA *(AN10476, *A. nidulans *IMPDH) were amplified using primer pairs BGHA168/BGHA169 and BGHA170/BGHA171. pHC3 was created by USER cloning these fragments into pHC2 following the USER cloning method previously described [18,20]. All plasmids were propagated in *Escherichia coli *strain DH5α. All primers used in this study are listed in Table 2.

**Table 2:** List of primers

Name	Sequence (5' → 3')
BGHA236 HC	ATGCCIATYNCCRMCGGIGAYKC
BGHA246 HC	CRGCCTTCTTRTCCTCCATGG
BGHA240 HC	ATGGTCGADRTYCWGGAYTAYACC
BGHA241 HC	GARGCRCCRGCGTTMTTG
BGHA343	GAGCGYATGARYGTYTAYTTCA
BGHA344	GTGAACTCCATCTCRTCCATACC
BGHA70	TTAACACAATTGCGCGGTTTTTTGGGGTAGTCATC *MfeI*
BGHA71	TTAACACCTGCAGGCGCGGTTTTTTGGGGTAGTCATC *SbfI*
BGHA125	TTAACAGGGTACCAAGTCAATTTTCACCAATCAAGC *KpnI*
BGHA132	TGGTATGCGATCGCGTCAGAGTCAAACAAAGCCAGA *AsiSI*
BGHA168	GGGTTTAAUACAGACGAAAGGGTTGTTGG
BGHA169	GGACTTAAUGTCTCTATCAGGACACGCAGA
BGHA170	GGCATTAAUTGGCTTTCTTTTCGTTTCTTG
BGHA171	GGTCTTAAUTGCTTCTGCAATTTCGACAC
BGHA98	GGTTTCGTTGTCAATAAGGGAA
BGHA256 HC	CATGGAGGGCTTCCAGAATA
BGHA255 HC	TTTTGCTGTGCTGTAGTCGTG
BGHA225	CCAGTTATCTGGGCAAACCAAAAG

### *A. nidulans *strain construction

Protoplasting and gene-targeting procedures were performed as described previously [21,22]. 5 μg pHC3 was digested with NotI to liberate the gene targeting substrate, which was used for transformation of NID3 [23]. Transformants containing the desired gene targeting event were verified by PCR with primer-pairs BGHA98/BGHA256HC and BGHA255HC/BGHA225 using *Taq*-polymerase (Sigma-Aldrich) on genomic DNA obtained from streak purified transformants extracted using the FastDNA^® ^SPIN for Soil Kit (MP Biomedicals, LLC).

### MPA treatment of fungi

Spores from *A. nidulans *NID191 and *A. nidulans *NID495 were harvested. 10-fold dilution series was performed on freshly made MM-plates with 0, 5, 25, 100, 200 μg MPA/ml (Sigma). All plates contained 0.8% (v/v) methanol. Relative growth of the strains was assessed by visual inspection.

### Degenerate PCR

An alignment with the DNA sequence (including introns) of the genes encoding *P. brevicompactum *IMPDH-B, *A. nidulans *IMPDH-A, *P. chrysogenum *IMPDH-A, *P. chrysogenum *IMPDH-B and *Aspergillus fumigatus *IMPDH-A was created by using ClustalW [24]. The ClustalW algorithm was accessed from the CLC DNA workbench 5 (CLC bio, http://www.clcbio.com/) with the following parameters: 'gap open cost = 20.0', 'gap extension cost = 1.0', and 'end gap cost = free'. The alignment was used to design degenerate primers to amplify either IMPDH-A like genes (BGHA236HC/BGHA246HC) or IMPDH-B like genes (BGHA240 HC/BGHA241 HC). The primer-set BGHA343/BGHA344 was used to amplify the β-tubulin sequence. Genomic DNA from *P. brevicompactum *IBT 23078 and four other fungi from *Penicillium *subgenus *Penicillium *were extracted using the FastDNA^® ^SPIN for Soil Kit (MP Biomedicals, LLC). Touch-down PCR was carried out using Phusion polymerase (Finnzymes) and the following program. An initial denaturation cycle at 98°C for 2 min; followed by 35 cycles at 98°C for 30 s, an annealing step ranging from 61°C (first cycle) to 54°C (last cycle) for 30 s, and extension at 72°C for 45 s. PCR mixture was made according to the manufacture's instructions. PCR products generated by degenerate PCR were purified from agarose gels using illustra™ DNA and Gel band purification kit (GE Healthcare). Sequencing of purified PCR products was performed by StarSeq (Germany).

### Cladistic analysis

BLASTx search was performed with standard settings: 'blastp algorithm', 'expect threshold = 10', 'word size = 3', 'max matches in query range = 0', 'matrix = BLOSUM62', 'gap open cost = 11', 'gap extension cost = 1', and no filters were used. Alignment of DNA coding regions were performed with ClustalW [24] as implemented in the CLC DNA workbench 5 (CLC bio, http://www.clcbio.com/) and by using the following parameters: 'gap open cost = 20.0', 'gap extension cost = 1.0', and 'end gap cost = free'. A cladogram was constructed with the same software using the neighbour-joining method and 1000 bootstrap replicates [25]. The DNA sequence of IMPDH and β-tubulin from selected fungi with sequenced genome were retrieved from NCBI. These included IMPDH sequence from *A. nidulans *[GenBank:ANIA_10476], *Aspergillus terreus *[GenBank:XM_001218149], *Aspergillus niger *[GenBank:XM_001391855], *P. chrysogenum *putative IMPDH-A coding gene, [GenBank:XM_002562313], putative IMPDH-B coding gene [GenBank:XM_002559146], *P. marneffei *[GenBank:XM_002151867]. β-tubulin sequences from *A. nidulans *[GenBank:XM_653694], *A. terreus *[GenBank:XM_001215409], *A. niger *[GenBank:XM_001392399], *P. chrysogenum *[GenBank:XM_002559715] and *P. marneffei *[GenBank:XM_002151381]. The MPA gene cluster sequence from *P. brevicompactum*, which contains the IMPDH-B sequence (*mpaF*) is available from GenBank under accession number [GenBank:HQ731031].

### Protein alignment

Amino acid sequences were aligned with ClustalW [24] as implemented in the CLC DNA workbench 5 (CLC bio, http://www.clcbio.com/) by using the following parameters: 'gap open cost = 20.0', 'gap extension cost = 1.0', and 'end gap cost = free'.

### Nucleotide sequence accession numbers

All sequences obtained via degenerate PCR were submitted to GenBank (Table 1). *Penicillium bialowiezense β-tubulin *[GenBank:JF302653], putative IMPDH-A coding gene [GenBank:JF302658], putative IMPDH-B coding gene [GenBank:JF302662], *P. brevicompactum β-tubulin *[GenBank:JF302653], *imdA *[GenBank:JF302657], *Penicillium carneum β-tubulin *[GenBank:JF302650], putative IMPDH-A coding gene [GenBank:JF302656], putative IMPDH-B coding gene [GenBank:JF302660], *Penicillium paneum β-tubulin *[GenBank:JF302651], putative IMPDH-A coding gene [GenBank:JF302654], putative IMPDH-B coding gene [GenBank:JF302661], *Penicillium roqueforti β-tubulin *[GenBank:JF302649], putative IMPDH-A coding gene [GenBank:JF302655], putative IMPDH-B coding gene [GenBank:JF302659].

## Authors' contributions

KRP, UHM, BGH and TBR conceived the study. BGH designed the experiments. BGH, HJG, CSK and JBN carried out the research. JCF contributed to the design of experiments and provided expertise in mycology. BGH and HJG prepared the first draft of the manuscript. UHM and KRP contributed to the experimental design and preparation of the manuscript. All authors were involved in the revision of the draft manuscript and have agreed to the final content.
